# Development of a Deep Learning-Based Decision Framework for Optimal Process Parameter Selection in Metal Additive Manufacturing

**DOI:** 10.3390/s26041124

**Published:** 2026-02-09

**Authors:** Min Seop So, Duck Bong Kim, Duncan Kibet, Jong-Ho Shin

**Affiliations:** 1 Department of Industrial Engineering, Chosun University, Gwangju 61452, Republic of Korea; sms3310@chosun.kr (M.S.S.); duncankibet90@gmail.com (D.K.); 2 School of Environmental, Civil, Agricultural, and Mechanical Engineering, University of Georgia, Athens, GA 30602, USA; dbkim@uga.edu

**Keywords:** wire + arc additive manufacturing, surface roughness, deep neural network, arc welding

## Abstract

Conventional subtractive manufacturing methods, such as cutting, often result in material waste and limitations in geometric complexity. To address these challenges, Wire Arc Additive Manufacturing (WAAM), in which components are built through successive weld bead deposition, has attracted increasing attention across various industrial fields. However, WAAM-fabricated components typically exhibit significant surface irregularities, necessitating additional post-processing that reduces overall productivity. Improving productivity therefore requires effective control and optimization of deposition parameters. This task is particularly challenging in multilayer WAAM processes, as the geometry of previously deposited layers varies with operating conditions. To address this challenge, this study proposes an AI-based framework for controlling surface roughness by rapidly identifying near-optimal process parameters in response to evolving bead geometry. A large-scale simulation dataset was generated by applying a pre-trained deep neural network (DNN) surface roughness predictor to one million bead geometry variations under 72 process parameter combinations. The resulting optimal parameter labels were used to train a classification model that recommends process conditions based on the current bead geometry. Model performance was evaluated using predictor-estimated surface roughness values, achieving *Weighted Precision*, *Recall*, and *F*1-*score* of 0.98, with an average AUC of 0.977. Five previously generated WAAM specimens were used for comparative analysis between AI-recommended and conventional process conditions using the previously developed and validated surface roughness prediction model, rather than direct physical measurements. This predictor-based feasibility analysis showed that AI-recommended conditions consistently reduced the predicted surface roughness, indicating the potential of AI-driven process optimization to improve surface quality in WAAM and reduce reliance on post-processing.

## 1. Introduction

Wire Arc Additive Manufacturing (WAAM) has emerged as a promising metal additive manufacturing (AM) technology for industrial applications such as mold manufacturing, medical devices, automotive, and aerospace components. WAAM employs an electric arc as the heat source to deposit metal wire layer by layer onto a substrate. Compared with conventional subtractive manufacturing, WAAM offers advantages including higher material utilization, lower equipment cost, and the capability to fabricate large-scale and geometrically complex components that are difficult to achieve using traditional manufacturing methods [1,2,3]. Furthermore, WAAM requires lower equipment investment than many other AM technologies [4] and offers high material utilization with elevated deposition rates, making it well suited for applications involving expensive materials [5,6]. These advantages make WAAM a compelling alternative to conventional manufacturing, particularly for the fabrication of large-scale metallic components in industries such as shipbuilding and aerospace.

**Figure 1:**
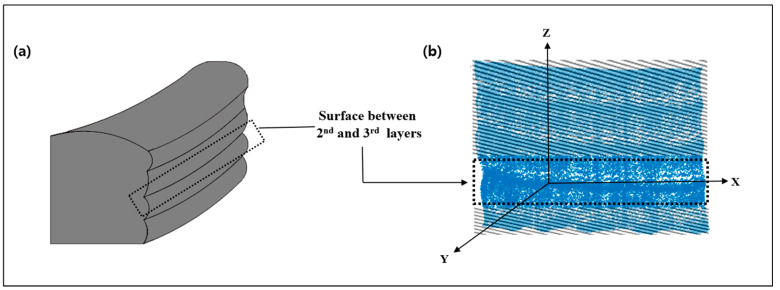
(**a**) WAAM product surface between the second and third layers. (**b**) Reconstruction of WAAM product between the second and third layers using a CMM [7].

Despite these advantages, poor surface quality remains a major barrier to the industrial adoption of WAAM. Unlike conventional manufacturing, where surface roughness is evaluated at the micro scale, WAAM components exhibit macro-scale roughness caused by the layer-by-layer deposition process (Figure 1). In this study, macro-level surface roughness is defined as the deviation from the center plane between two consecutive layers, following our previous work [7].

The high heat input inherent to arc welding often results in poor surface roughness in WAAM, which degrades mechanical properties such as tensile strength and fatigue resistance and necessitates extensive post-processing. These finishing operations increase manufacturing time, cost, and material waste, particularly in performance-critical applications such as aerospace and medical components. Consequently, accurate prediction of surface roughness based on process parameters and prior-layer bead geometry during deposition is essential. In our previous work, a deep neural network (DNN) model trained on experimental GMAW–WAAM data successfully predicted interlayer surface roughness using wire feed rate, travel speed, and bead geometry features, achieving high predictive accuracy (MAPE = 1.93%, RMSE = 0.03, R = 0.97) [7]. Although the model predicts surface roughness under varying process parameters, it does not provide optimal parameter recommendations, and parameter selection therefore remains largely trial-and-error. While prior studies have explored process optimization, many focus on post-processing strategies rather than in situ control. Reducing interlayer roughness during deposition can decrease subsequent material removal, tool wear, and cycle time, thereby lowering manufacturing cost and scrap [8]. Moreover, internal surfaces with complex geometries, such as deep channels and multi-intersection passages, are often inaccessible to conventional finishing and measurement tools [9]. As described, poor as-built surface roughness increases post-processing time and cost. Therefore, optimizing process parameters in real-time during deposition would be a more effective approach since it enables partial control of surface roughness in situ and thereby reduces the extent and cost of subsequent post-processing.

Xiong et al. [10] investigated the optimal process parameter for improving surface roughness and found that a lower interlayer temperature improving surface quality when other parameters remained constant. They further concluded that combining low wire feed and travel speeds produced the best surface quality in GMAW-based AM. Wang et al. [11] demonstrated that controlling the welding current for each layer section could effectively enhance the surface quality of the variable polarity gas tungsten arc welding (VP-GTAW) process. Similarly, Dinovitzer et al. [2] examined the effects of current variations on surface roughness while welding Hastelloy X alloy and concluded that increasing the welding current significantly improved surface finishing. Hanif et al. [12] conducted a finite element and gray relational analysis to optimize bead geometry in TIG welding of mild steel, identifying welding current as the most significant factor influencing thermal stress and temperature distribution. Previous studies, including our earlier work [7], have shown that various process parameters affect surface roughness, and this study also focuses on two of them (i.e., feed rate and travel speed) as control variables.

Various studies have confirmed that multiple process parameters directly influence surface roughness. Several analytical models have been proposed to enhance the surface quality of multi-bead overlapping processes [13,14,15,16,17,18]. Wurilaixi et al. [13] developed an overlapping model to optimize process parameters and achieved smooth surfaces in micro-plasma arc welding. Their study constructed an improved overlapping model using two key parameters: overlapping ratio and scan spacing. Lam et al. [16] introduced an overlapping model for tool-path generation to minimize the valleys between adjacent beads. Earlier models primarily considered geometric bead areas; however, they neglected the spreading behavior of weld beads, which is critical for multi-bead deposition. Yongzhe et al. [14] addressed this limitation by proposing an enhanced bead-overlapping model that incorporated bead-spreading characteristics to significantly improve the surface flatness of WAAM-produced multilayer components.

In addition to geometric models, statistical and AI-based optimization approaches for refining surface quality have been explored. Lee [5] utilized Gaussian process regression (GPR) to model the uncertainty in multiple WAAM process parameters, including wire feed rate, travel speed, and interpass time, to optimize surface quality. Calignano et al. [19] applied Taguchi-based optimization to improve aluminum part surface roughness in direct metal laser sintering (DMLS), selecting laser power, scan speed, and hatching distance as key parameters. Masood et al. [20] developed a mathematical algorithm for optimizing part orientation in fused deposition modeling to enhance surface quality. Similarly, Ning et al. [21] implemented a neural network with backpropagation to determine the optimal DMLS process parameters and demonstrated improved roughness in customized parts. Some researchers have focused on real-time control strategies for enhancing surface quality. Dong et al. [22] introduced a Gaussian Process Regression surrogated Bayesian optimization algorithm (GPRBOA) for real-time weld quality control in GTAW. Wu et al. [23] proposed an oscillating wire transfer technique for improving surface smoothness. Xiong et al. [24] developed an image-processing-based monitoring and control system integrated with a segmented neuron self-learning controller to reduce material waste and enhance surface quality in GMAW-based AM. Li et al. [25] proposed a machine learning-based approach using Particle Swarm Optimization-optimized support vector machine to predict and optimize process parameters in WAAM, significantly reducing dependence on trial-and-error experiments. Their model achieved high predictive accuracy and demonstrated up to 11.47% reduction in energy consumption through validation experiments. Dharmawan et al. [26] applied reinforcement learning (RL) to control process parameters in robotic WAAM, particularly for multilayer multi-bead deposition. Hybrid additive-subtractive manufacturing approaches have also been explored [18,27,28,29]. Although hybrid manufacturing systems can improve surface finish through integrated post-processing, they do not eliminate material waste or reduce dependence on post-processing due to their inherent process characteristics.

Previous studies have demonstrated that process parameters strongly influence surface quality in additive manufacturing; however, most existing approaches rely on geometric overlapping models or statistical optimization and do not explicitly account for the influence of prior-layer bead geometry on subsequent layers. In multilayer deposition, the stair-step effect causes surface quality to depend on both prior bead geometry and process conditions, underscoring the need for geometry-aware process parameter optimization.

To address this gap, this study proposes an AI-based decision-making framework for layer-by-layer optimization of WAAM process parameters by integrating an experimentally validated surface roughness prediction model. The proposed approach learns a geometry-conditioned policy offline and enables real-time selection of wire feed rate and travel speed based on prior-layer bead geometry, without requiring online exploration as in reinforcement learning or Bayesian optimization methods. Experimental validation using a GMAW–WAAM system confirms the effectiveness of the proposed framework in improving surface quality.

The remainder of this paper is organized as follows. Section 2 describes the proposed optimization framework and simulation-based data generation. Section 3 presents experimental results and performance evaluation. Section 4 concludes the paper and discusses future research directions.

## 2. Proposed Methodology

This study aims to overcome the limitations of previous research by proposing an AI-based decision-making system utilizing a deep learning optimization model to determine the optimal process parameters for minimizing surface roughness in WAAM. Unlike conventional approaches that rely on predefined process parameters or empirical trial-and-error adjustments, the proposed framework dynamically predicts the most effective process parameters based on the real-time bead geometry information of previous layer. This section details the methodology, which comprises three major stages: simulation-based data generation, optimal process parameter extraction, and AI-based decision-making model development.

### 2.1. Data Generation Using Simulation

**Figure 2:**
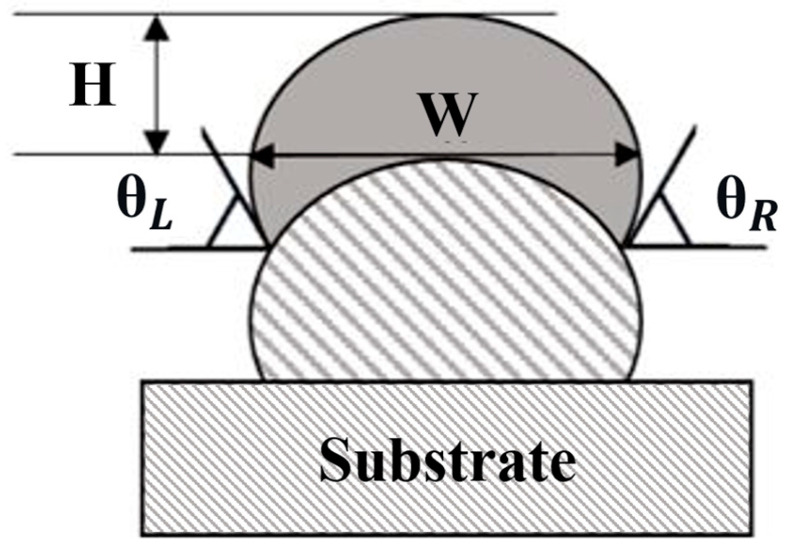
Schematic diagram of bead shape [7].

Real-world data collection is costly and time-consuming; therefore, the authors used a simulation-based approach to generate a diverse dataset for AI model training. In the previous study of authors, 81 bead geometry samples were collected from 27 fabricated WAAM walls. Briefly, the experimental setup comprising a 6-axis robot (Fanuc ArcMate 120iC (FANUC Corporation, Rochester Hills, MI, USA)) for motion control, a GMAW-CMT system (Fronius TPS 400i with WF 25i Robacta Drive torch (Fronius International GmbH, Wels, Austria)) for low-heat, stable deposition, and a portable CMM (Hexagon Romer Arm 7525SIE (Hexagon Manufacturing Intelligence, North Kingstown, RI, USA) to acquire 3D surface profiles; feed rate and travel speed were commanded by the system controllers, and full sensing/collection procedures are detailed in [7]. Five key parameters characterize each bead geometry: layer number, bead height, bead width, left angle (θ_*L*_), and right angle (θ_*R*_) (see Figure 2).

Figure 2 illustrates the bead geometry parameters used as input features for surface roughness prediction and process parameter optimization. The bead width (W) and height (H) describe the geometric dimensions of the deposited bead, while the left and right angles ($\theta_{L}$ and $\theta_{R}$) characterize the sidewall inclination formed by overlapping layers. Together with the layer number, these parameters represent the deposition state of the previously formed layer and strongly influence the stair-step effect and interlayer surface roughness in multilayer WAAM. Rather than exhibiting a single set of optimal values, these geometric parameters interact nonlinearly with process parameters such as wire feed rate and travel speed. Consequently, optimal surface roughness is achieved not by fixed geometric thresholds but by selecting appropriate process parameters conditioned on the current bead geometry. This geometry-conditioned relationship forms the basis of the proposed AI-based decision-making framework.

The surface roughness prediction model employed in this study was developed and experimentally validated in our previous work. Briefly, the predictor is a deep neural network comprising multiple fully connected layers with ReLU activation functions, trained to estimate interlayer surface roughness from bead geometry features (layer number, bead width, bead height, left angle, and right angle) together with process parameters (wire feed rate and travel speed). The model was trained using experimentally measured surface profiles obtained from WAAM-deposited thin-wall structures and demonstrated strong predictive performance, achieving a mean absolute percentage error (MAPE) of 1.93%, a root mean squared error (RMSE) of 0.03, and a correlation coefficient of 0.97 within the experimentally validated operating range. In the present study, this predictor is used as a surrogate model to estimate surface roughness under different process parameter combinations.

To expand the dataset and cover a broad spectrum of potential bead geometries, random sampling was used to generate one million unique combinations of bead geometry. These combinations were created by mixing geometric features such as bead width, bead height, and left/right angles derived from experimentally obtained data. The process parameters for GMAW–CMT deposition were identical to those used in our previous study [7] (see Table 1). Travel speed was varied from 30 to 33 cm/min (in 1 cm/min increments), and the feed rate was adjusted from 480 to 650 cm/min (in 10 cm/min increments), resulting in 72 unique process parameter combinations.

For each generated bead geometry, simulations were conducted using a pre-trained DNN-based surface roughness prediction model across 72 process parameter combinations. This simulation-based approach enabled surface roughness estimation under diverse process parameters, ultimately producing a comprehensive dataset for training the proposed AI-based decision-making model.

**Figure 3:**
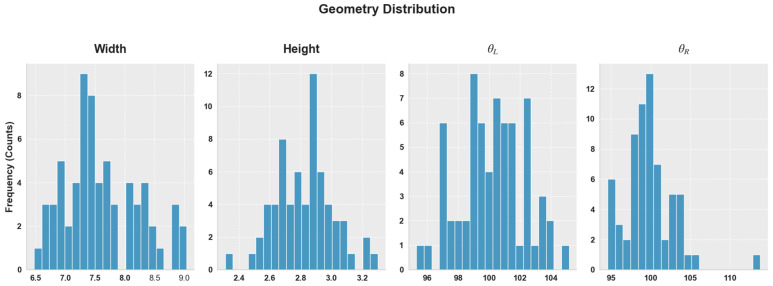
Distribution of the geometry.

In this study, the term “mixing” refers to a constrained random sampling procedure based on experimentally observed bead geometry distributions rather than arbitrary interpolation or extrapolation. Each geometric feature (layer number, bead width, bead height, left angle, and right angle) was independently sampled within the minimum and maximum ranges measured from the fabricated WAAM specimens. No extrapolation beyond the experimentally validated geometry space was performed. To ensure physical realism, only geometrically feasible combinations were retained, including positive bead width and height and sidewall angles consistent with stable bead formation observed in the experimental dataset. The ranges for each geometry were strictly defined based on the observed experimental limits: width (6.48~9.04 mm), height (2.31~3.30), left angle (95.30°~105.18°), and right angle (94.57°~113.81°). The specific distribution of these variables is illustrated in Figure 3, ensuring that the synthetic dataset spans a realistic parameter space. Consequently, the generated bead geometries represent physically plausible WAAM deposition states representative of real multilayer builds.

Among the fabricated 27 WAAM walls, 22 of them were used to generate the training dataset for generating one million bead-geometry variations, while the remaining five randomly selected a priori were reserved solely for validation. To estimate surface roughness under various process parameters, a pre-trained DNN-based simulation model was employed. Consequently, the training labels for these one million geometries are defined as pseudo-ground truth derived from the predictive model rather than direct physical measurements. Each of the one million bead geometry variations were evaluated with 72 different combinations of process parameters (feed rate and travel speed). The simulator predicted the resulting surface roughness for every bead geometry–parameter pair, thereby generating a large dataset that mapped bead geometries and process parameters to their corresponding surface roughness values. The structure of this dataset is illustrated in Table 2. The first column, labeled “Index”, denotes the sample number generated by the simulator. The second column indicates the layer number corresponding to the previously deposited layer, and the third through sixth columns contain bead geometry features specifically, left angle ($\theta_{L}$), right angle ($\theta_{R}$), width, and height. The remaining columns represent the predicted surface roughness by each of the 72 process parameter combinations. Among these, the parameter that yields the lowest surface roughness highlighted in bold red is defined as the optimal setting for that particular bead geometry.

### 2.2. Definition of Input Data and Output Data and Normalization

The dataset used in this study was generated using a simulation-driven approach, in which bead geometries were randomly sampled, and the optimal process conditions were determined based on predictive AI model [7]. The input data comprised bead geometry that were randomly generated to cover a wide range of possible deposition conditions. The output data represented the optimal combination of process parameters (feed rate and travel speed), resulting in the lowest prediction of surface roughness, as determined through a simulation-based evaluation of all feasible process parameter settings. Table 3 presents the structure of the dataset used for AI model training. The first column “Index” presents the unique identifier assigned to each bead geometry sample. The second column “Layer” indicates the deposition layer within the multilayer WAAM process. Bead geometries are influenced by previously stacked layers; therefore, this information helps contextualize variations in surface roughness. The third through sixth columns ($\theta_{L}$, $\theta_{R}$, Width, Height) define the bead geometry characteristics extracted from the experimental dataset and used as input variables. These inputs provide critical information on the deposition state of the previously stacked layer, which directly affects the subsequent layer’s surface roughness. The output data presented in the last column of Table 3 contain the optimal process parameter combination (feed rate and travel speed) selected for each bead geometry. These optimal process parameters were determined by systematically evaluating all 72 possible process parameter combinations for each bead geometry using a pretrained surface roughness prediction model [7]. The prediction model estimated the expected roughness for each parameter setting. The combination that resulted in the least roughness was selected as the optimal process parameters. Structuring the dataset in this manner enables the AI model to learn the relationship between bead geometry variations and the most effective process parameters, thus facilitating real-time decision-making in WAAM. The comprehensive dataset ensures that the trained model can dynamically predict the optimal process parameters based on bead geometry, thereby reducing the need for trial-and-error parameter tuning and improving the overall process stability and efficiency. In this study, the output process parameters correspond exclusively to wire feed rate and travel speed, which are encoded as 72 discrete parameter combinations in the output labels.

Each variable is normalized since the measured values had different ranges. It is aimed to reduce the influence of deviations caused by differences in the measurement range of each variable. Furthermore, normalization can reduce the learning time of machine learning models and prevent decreases in accuracy caused by heavy computation [30]. Table 4 presents the robust scaler normalization is used in this study.

### 2.3. DNN Model Development

**Figure 4:**
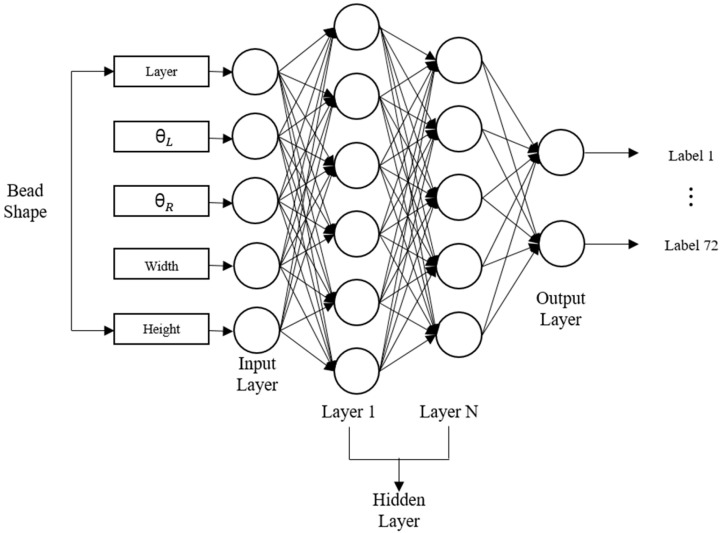
Schematic diagram of the deep neural network for the prediction of optimal process.

An artificial neural network is structured as single-layer perceptron, resulting in limitations when attempting to address nonlinear problems. A deep learning model has been proposed that can compensate for this drawback by utilizing multiple hidden layers and backpropagation. Recently, the DNN has been extensively applied across various domains and demonstrated impressive performance. Therefore, in this study, a DNN-based model is adopted. As a fundamental deep learning model, the DNN is structured with multiple hidden layers, providing advantages for comprehending the complex structure of large datasets and learning diverse nonlinear relationships. Figure 4 illustrates the proposed structure. The DNN structure can change depending on the hyperparameters (number of hidden layers, optimizer, and learning rate). Consequently, determining the optimal DNN structure requires trial and error.

### 2.4. Performance Measure

The dataset was partitioned into training, validation, and test sets to train the model properly and avoid overfitting. In this study, the dataset comprised 65% training data, 15% validation data, and 20% test data. The training set was used exclusively to train the model by optimizing its weights and identifying patterns, whereas the validation set assisted in tuning the hyperparameters and detecting potential overfitting by assessing the generalization capability of the model. Finally, a test set was reserved to evaluate the performance of the trained model under new process parameter settings. To mitigate the risk of overfitting and underfitting caused by class imbalance, the “stratify” option in the Scikit-learn library was used to ensure that the class distribution of the original dataset was preserved during the splitting process.

**Figure 5:**
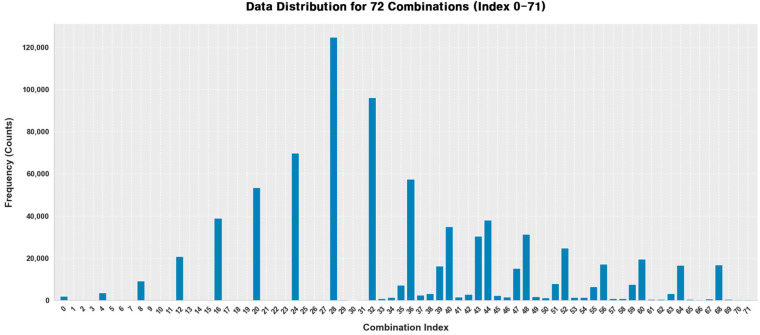
Class distribution of the 72 process parameter combinations arising from geometry-conditioned optimization.

Figure 5 shows the class distribution of the 72 process parameter combinations used in this study. As observed, the distribution is imbalanced, with certain parameter combinations occurring more frequently as optimal solutions depending on bead geometry. This imbalance arises naturally from the geometry-conditioned optimization process rather than from data collection bias. To address this issue and ensure fair evaluation across all classes, stratified data splitting and *Weighted Precision*, *Recall*, and *F*1-*score* metrics were employed.

*Weighted Precision*, *Recall*, and *F*1-*score* were selected as the primary metrics to evaluate the performance of the AI-based decision-making model for process parameter optimization in WAAM (see Equations (4)–(6)). These metrics were used to assess the capability of the model to predict the optimal process conditions accurately. This is crucial for achieving high surface quality while minimizing inefficiencies in the WAAM process. Furthermore, these metrics comprehensively evaluate classification accuracy by considering class imbalances in the dataset. In datasets with imbalanced class distributions, standard *Precision* and *Recall* may be misleading since majority classes dominate the overall evaluation. By applying class weighting, these metrics ensure a balanced assessment across all categories, preventing majority classes from disproportionately influencing the evaluation. *Weighted Precision* was used to calculate the *Precision* for each class individually and then averaged while accounting for the relative frequency of each class, ensuring a more representative accuracy measure across all classes. *Weighted Recall* and *F*1-*score* followed the same weighting approach, providing a balanced evaluation of the classification capability of the model. Higher values for these metrics indicate better classification performance. *Precision* quantifies the proportion of correctly identified optimal process conditions among all instances classified as optimal by the model and is calculated using the following formula:

(1) $$Precision=\;\frac{\mathrm{TP}}{\mathrm{TP}+\mathrm{FP}}$$

where TP (true positives) represents cases in which the model correctly predicted the actual optimal process condition, and FP (false positives) indicates cases in which the model incorrectly classified a suboptimal condition as optimal. A higher *Precision* value suggests that the model effectively minimizes false optimal predictions, thereby reducing the likelihood of selecting suboptimal process settings that may degrade surface quality.

(2) $$Recall=\frac{\mathrm{TP}}{\mathrm{TP}+\mathrm{FN}}$$

where FN (false negatives) represents the actual optimal process conditions that the model failed to identify. A higher *Recall* value indicates that the model successfully detected a larger proportion of true optimal process parameters, thereby ensuring that potentially high-quality settings were not overlooked.

*F*1-*score* balance *Precision* and *Recall* to provide a comprehensive assessment of the model’s classification performance and are calculated as follows:

(3) $$F1-score=\;2\times \frac{Precision\times Recall}{Precision+Recall}$$

A high *F*1-*score* signifies that the model effectively identifies the optimal process conditions while maintaining a low false-positive rate. This is particularly valuable in real-world WAAM applications, in which incorrect selection of process parameters can lead to increased surface roughness, additional post-processing requirements, or even structural defects in fabricated parts.

In imbalanced datasets, standard metrics can be misleading since majority classes dominate the evaluation. To address this issue, *Weighted Precision*, *Recall*, and *F*1-*score* are computed by averaging class-wise values according to sample frequency [31].

(4) $$Weighted\;Precision=\frac{\sum_{i=1}^{n}{precision}_{i}\times {count}_{i}}{Total\;count}$$

(5) $$Weighted\;Recall=\frac{\sum_{i=1}^{n}{recall}_{i}\times {count}_{i}}{Total\;count}$$

(6) $$Weighted\;F1-Score=\frac{\sum_{i=1}^{n}{f1}_{i}\times {count}_{i}}{Total\;count}$$

In the weighted formulations *precision_i_*, *recall_i_*, *f*1*_i_* denote the class-wise *Precision*, *Recall*, and *F*1-*score* for class *recall_i_*, respectively; *count_i_* represents the number of samples in *class_i_*; and *Total count* is the total number of samples. This ensures that less frequent classes contribute proportionally to the overall evaluation, preventing majority classes from dominating the metrics. The classification performance of the trained DNN-based decision-making model was assessed using weighted evaluation metrics. This ensured that the model could accurately predict the optimal process parameters for WAAM, thereby reducing the need for manual tuning and trial-and-error adjustments. Robust *Precision*, *Recall*, and *F*1-*score* evaluations were used to confirm that the AI-based approach can reliably optimize the process conditions, contributing to consistent surface quality improvements and overall process efficiency.

### 2.5. Experimental Results

Table 5 presents the structure and performance results of the DNN model developed for process parameter optimization in WAAM. The table details the key architectural components, training techniques, and evaluation metrics used to assess the effectiveness of the proposed model in predicting the optimal process conditions for minimizing surface roughness.

An AI-based decision-making model for process parameter optimization in WAAM was implemented using DNN architecture. The model was designed as a multilayer feedforward network comprising multiple fully connected layers to learn the complex relationships between bead geometry and optimal process parameters. The input layer of the model used five key features extracted from the bead geometry of the previous layer, whereas the output layer comprised 72 classes representing different feed rate and travel speed combinations. The Softmax activation function was applied to the output layer to enable the optimal process conditions to be classified. The hidden layers of model were structured with progressively increasing and decreasing neuron counts to enhance learning capacity while simultaneously preventing overfitting. Each layer employed the ReLU activation function to introduce nonlinearity, thereby facilitating the ability of the model to capture intricate patterns in the data. Furthermore, L2 regularization was applied to mitigate overfitting, and an Adam optimizer with a learning rate of 0.001 was used for efficient convergence.

Early stopping, model checkpointing, and learning rate reduction techniques were applied to further optimize the training. Early stopping was configured to stop the training when the validation accuracy did not improve for 300 consecutive epochs, whereas model checkpointing ensured that the best-performing model (in terms of validation accuracy) was saved. A learning rate scheduler was used to reduce the learning rate by a factor of 0.1 if no improvement was observed in validation accuracy for 50 epochs. The final trained model demonstrated high accuracy in predicting optimal process parameters, achieving a *Weighted Precision* of 0.98, *Weighted Recall* of 0.98, and *Weighted* *F*1-*score* of 0.98. These results indicate that the model effectively identifies the best process conditions for minimizing surface roughness while maintaining a balanced classification performance across all process parameter classes. The high *F*1-*score* confirms that the model maintained a strong balance between *Precision* and *Recall*, thereby minimizing false positives and negatives. The optimized DNN system uses bead geometry to adjust parameters in real time, improving WAAM surface quality and reducing manual tuning. An additional evaluation was conducted using the receiver operating characteristic (ROC) curve and area under the curve (AUC) to further validate the performance of the trained model in predicting the optimal process parameters.

**Figure 6:**
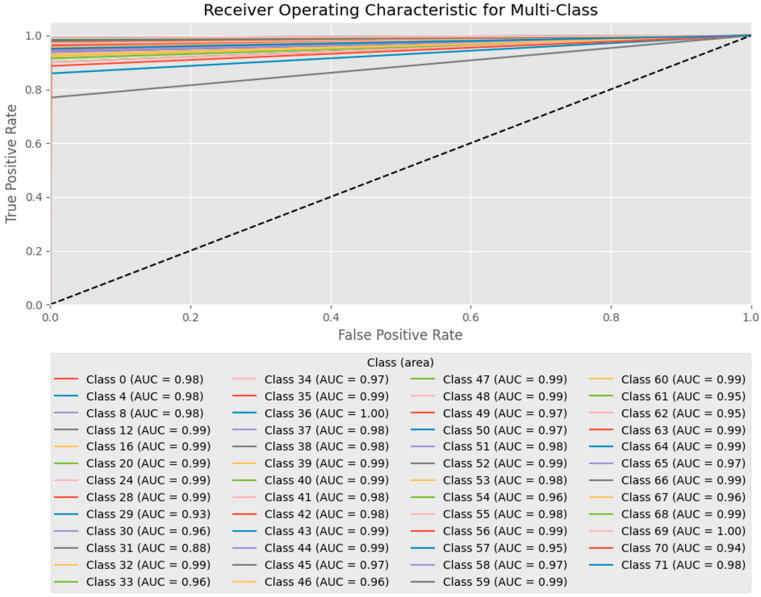
Multi-class ROC curves and AUC scores for optimal process condition classification.

The ROC curve is a graphical representation that illustrates the diagnostic ability of a multiclass classification model by plotting the true positive rate (sensitivity) against the false positive rate (1-specificity) across the classification threshold settings. The AUC quantifies the classifier’s ability to distinguish between classes. Values closer to 1.0 indicate stronger classification performance, whereas values around 0.5 suggest random guessing. For multiclass classification, the mean AUC value was computed as the average AUC across all classes, providing a robust metric for evaluating model performance while accounting for class imbalances and variations across test instances. Higher mean AUC values suggest that the model can accurately predict optimal process conditions and ensure better decision-making reliability. For the deep learning model trained in this study, ROC curves were generated for all 72 process condition classes, and their respective AUC values are presented in Figure 6. The model exhibited consistently strong classification performance across most classes. The overall mean AUC for the model was 0.977, indicating that the classifier could successfully differentiate between various process parameter settings and accurately predict the optimal conditions for minimizing surface roughness in WAAM. The high mean AUC value underscores the model’s generalization ability and robustness in handling diverse process conditions, ensuring reliable decision-making even in complex manufacturing environments. These results validate the effectiveness of the proposed AI-driven decision-making system in optimizing the process parameters for WAAM. The strong predictive performance of the model demonstrates its potential for significantly reducing process variability, enhancing surface quality, and minimizing reliance on trial-and-error parameter tuning, thereby improving industrial application efficiency.

## 3. Analysis of the Results

It should be noted that the surface roughness values for the DNN-recommended conditions reported in Table 6 were obtained using the previously validated surface roughness prediction model [7], rather than direct physical measurements, and therefore represent a predictor-based evaluation rather than a fully independent experimental validation.

It is noted that the absolute reductions in surface roughness reported in Table 6 are modest in magnitude. These values represent predictor-estimated roughness at the individual layer level rather than direct physical measurements and are therefore subject to the inherent uncertainty of the surface roughness prediction model. Consequently, the layer-wise comparisons in Table 6 are interpreted as a feasibility-level indication of the potential benefit of geometry-aware, AI-recommended process parameter selection. To provide a more robust quantitative assessment, specimen-level surface roughness statistics and formal significance testing are presented separately using aggregated experimental results.

The observed reduction in surface roughness under the DNN-recommended process conditions is consistent with prior studies reporting that appropriate adjustment of wire feed rate and travel speed can significantly influence bead geometry and interlayer surface quality in WAAM and arc-based additive manufacturing processes [10,11,25]. Similar trends have been reported in machine learning-based process optimization studies, where data-driven parameter selection reduced surface irregularities compared with fixed or empirically selected conditions [24,26].

Comparing conventional and DNN-recommended process conditions highlights the effectiveness of AI-driven optimization in reducing surface roughness. Five specimens (1st, 9th, 10th, 14th, and 22nd) were selected from 27 fabricated components and analyzed to validate the proposed model. The model was evaluated by comparing the applied process parameters and resulting surface roughness between the conventional and AI-optimized approaches. The DNN-recommended process parameters were validated using the surface roughness prediction model developed in our previous study, which was trained on experimental data collected from WAAM-deposited structures. This predictive model estimates the expected surface roughness based on bead geometry and process conditions, ensuring that AI-suggested parameters lead to meaningful improvements in surface quality.

For the first specimen, the second layer exhibited bead geometry with a left angle ($\theta_{L}$) of 102.11°, right angle ($\theta_{R}$) of 101.94°, width of 6.95 mm, and height of 2.06 mm. When depositing the third layer, the conventional process applied a travel speed of 31 cm/min and a wire feed rate of 560 cm/min, resulting in bead geometry of $\theta_{L}$ = 100.98°, $\theta_{R}$ = 99.87°, width of 6.91 mm, and height of 3.73 mm. Similarly, for depositing the fourth layer, the process parameters were adjusted to 30 cm/min and 480 cm/min, yielding a bead shape of $\theta_{L}$ = 105.3°, $\theta_{R}$ = 101.8°, width of 7.74 mm, and height of 2.89 mm. For the fifth layer, the process conditions were maintained at 30 and 480 cm/min. Under these conventional settings, the total surface roughness from the second through fifth layers was 3.255 µm. Conversely, when using the DNN-recommended process parameters, the third layer was deposited at 30 and 540 cm/min, the fourth layer at 30 and 570 cm/min, and the fifth layer at 30 and 570 cm/min. By applying these optimized conditions, the final surface roughness was reduced to 3.133 µm, which reflected an improvement over the conventional process.

To further evaluate the practical effectiveness of the proposed framework, a specimen-level comparative analysis was conducted between existing process parameters and AI-recommended conditions using five reserved validation specimens (see Table 7). Surface roughness values were aggregated across layers for each specimen, and the results were quantified using the mean reduction, percentage improvement. Under existing conditions, the mean surface roughness was 3.529 µm, whereas the AI-recommended conditions achieved a reduced mean roughness of 3.067 µm, corresponding to an average reduction of 0.462 µm and a 13.1% improvement in surface quality. A paired t-test was performed to assess statistical significance, yielding a t-statistic of 3.303 and a *p*-value of 0.0299. Since the *p*-value is below the 0.05 significance level, the reduction in surface roughness achieved through AI-driven optimization is statistically significant.

These findings align with recent AI-assisted and learning-based WAAM control studies, which demonstrated that geometry-aware or state-dependent parameter selection improves surface quality and reduces reliance on post-processing compared with conventional rule-based approaches [22,25,26].

The authors used this model to predict the optimal process parameters that minimize surface roughness, thereby confirming that the AI-based decision-making system can effectively identify deposition conditions for improved surface quality. These results demonstrate the feasibility of the proposed DNN-based decision-making framework in recommending process parameters that are predicted to improve surface quality under experimentally observed bead geometries. This demonstrates the practical applicability of deep learning for optimizing process conditions to achieve higher-quality AM. It should be noted, however, that the decision-making model was primarily trained on simulation-generated data based on a pre-trained predictor. As a result, potential biases from the predictor may propagate into the optimization process. While the present study demonstrates feasibility, additional experimental validation is required to confirm generalization under real WAAM conditions.

## 4. Conclusions and Future Research

This study proposed an AI-based decision-making framework for optimizing Wire Arc Additive Manufacturing (WAAM) process parameters with the objective of minimizing interlayer surface roughness. Building on a previously developed and experimentally validated deep neural network (DNN) surface roughness predictor, a large-scale simulation dataset consisting of one million bead geometry variations evaluated under 72 process parameter combinations was generated. Using this dataset, a DNN-based classification model was trained to recommend optimal wire feed rate and travel speed conditioned on the bead geometry of the previously deposited layer.

The proposed decision-making model demonstrated strong predictive performance, achieving *Weighted Precision*, *Recall*, and *F*1-*score* of 0.98 and an average area under the curve (AUC) of 0.977. These results indicate that the model can reliably identify near-optimal process parameter combinations across a wide range of bead geometries. Unlike conventional approaches that rely on fixed parameters or empirical trial-and-error adjustments, the proposed framework enables geometry-aware, layer-by-layer process parameter selection during WAAM deposition.

The primary novelty of this work lies in its evolution from a purely predictive model to an active decision-making system. While previous learning-based strategies often stop at predicting surface quality or monitoring bead geometry, our framework utilizes these inputs to autonomously recommend the optimal combination of process parameters (wire feed rate and travel speed) based on the observed bead geometry. By directly mapping geometry variations to optimized actions, the model bypasses the need for human intervention or secondary rule-based logic, providing a more advanced, end-to-end optimization architecture for autonomous manufacturing.

Furthermore, a key advantage of this framework is its scalability to higher-dimensional parameter spaces. While the present work is primarily focused on a two-parameter optimization problem (wire feed rate and travel speed) to demonstrate the fundamental feasibility of the approach, the total number of combinations grows exponentially as the number of process parameters and their respective ranges increase. In such cases, an exhaustive search becomes computationally prohibitive. In such high-dimensional spaces, the proposed decision model potentially integrated with strategies like Design of Experiments (DoE) serves as an efficient optimization tool to navigate complex parameter landscapes and identify optimal conditions without the need to evaluate the entire population.

Experimental verification was conducted using five fabricated WAAM thin-wall specimens that were excluded from the training dataset. For these specimens, surface roughness obtained under conventional working conditions was compared with that obtained under AI-recommended process conditions. The experimental results showed that the AI-recommended parameters consistently reduced surface roughness relative to conventional settings, demonstrating the practical applicability of the proposed approach in real WAAM operations.

Despite these promising results, several limitations remain. The optimization labels used to train the decision-making model were derived from a surrogate surface roughness predictor, and any residual modeling bias may propagate into the recommended parameters. In addition, equipment-specific variations across shop floors, such as differences in power source dynamics, torch geometry, and sensor calibration, may limit direct model transfer between machines operating under nominally identical settings. To address these challenges, the proposed framework can be coupled with on-machine sensing and initialized using the trained weights, followed by brief fine-tuning with a small site-specific dataset through transfer learning or domain adaptation.

Furthermore, while the proposed framework successfully improves surface quality on a layer-by-layer basis, it does not explicitly account for the cumulative influence of process parameters on long-term bead geometry evolution across multiple layers. Deposition parameters affect not only the surface roughness between consecutive layers but also the overall geometry evolution of the build. In future implementations, additional process variables such as interlayer temperature, arc voltage, torch angle, and shielding gas flow rate can be incorporated into the state and action spaces of the proposed framework, provided that appropriate sensing, actuation, and training data are available. Future research will therefore focus on integrating surface roughness prediction, bead geometry estimation, and reinforcement learning into a closed-loop control system. Such a multistep prediction and decision-making framework would enable real-time parameter adaptation and support fully autonomous, self-optimizing WAAM processes, thereby further enhancing *Precision* and efficiency for industrial applications.

## Figures and Tables

**Table 1:** Combination of dynamic process parameters.

**Combination No.**	Feed Rate	**Travel Speed**
1	480	30
2	480	31
︙	︙	︙
71	650	32
72	650	33

**Table 2:** Simulated Surface Roughness Results for Each Bead Geometry and Process Parameter Combination.

Index	Layer	θ*_L_* (°)	θ*_R_* (°)	Width (mm)	Height (mm)	Parameter Combination (Feed Rate, Travel Speed)	Surface Roughness
1	4th	102.08	99.84	7.54	2.54	1 (480, 30)	1.227
						2 (480, 31)	1.245
						︙	︙
						**36 (570, 30)**	**1.051**
						72 (650, 33)	1.199
︙	︙	︙	︙	︙	︙	︙	︙
10,000,000	4th	99.58	100.10	7.37	3.09	1 (480, 30)	1.111
						2 (480, 31)	1.127
						︙	︙
						**32 (560, 30)**	**1.047**
						72 (650, 33)	1.252

**Table 3:** Input and output structure.

Input Data						Output Data
Index	Layer	θ*_L_* (°)	θ*_R_* (°)	Width (mm)	Height (mm)	Parameter Combination (Feed Rate, Travel Speed)
1	4th	102.08	99.84	7.54	2.54	36 (570, 30)
2	2nd	100.38	103.8	6.98	2.64	16 (520, 30)
︙	︙	︙	︙	︙	︙	︙
999,999	3rd	97.08	98.76	8.07	2.89	43 (580, 33)
10,000,000	4th	99.58	100.10	7.37	3.09	32 (560, 30)

**Table 4:** Data Normalization.

Input Data						Output Data
Index	Layer	θ*_L_* (°)	θ*_R_* (°)	Width (mm)	Height (mm)	Parameter Combination (Feed Rate, Travel Speed)
1	0.5	0.63	0.04	0.1	−1.3	36 (570, 30)
2	−0.5	0.02	1.26	−0.65	−0.9	16 (520, 30)
︙	︙	︙	︙	︙	︙	︙
999,999	0	−1.17	−0.29	0.82	0.11	43 (580, 33)
1,000,000	0.5	−0.27	0.12	−0.13	0.9	32 (560, 30)

**Table 5:** Hyperparameters and evaluation metrics of the proposed DNN model.

Model	Model Parameters		Result (Weighted)		
**DNN**	**Layer**	(Number of input variables (5)), 1024, 512, 256, 128, 64, 32, 64, 128, 256, 512, 1024, 512, 256, 128,64, number of output variables (72))	**Precision**	**Recall**	**F1-Score**
	**Activation** **Function**	Dens Layer: ReLU Output Layer: Softmax	0.98	0.98	0.98
	**Optimizer**	Adam			
	**Learning Rate**	0.001			
	**Loss Function**	Sparse Categorical Cross entropy			
	**Batch Size**	1024			
	**Number of Epochs**	200,000			
	**Regularization**	L2 (λ = 0.001)			
	**Early Stopping**	Patience = 300 epochs			
	**Learning Rate Reduction**	Factor = 0.1, Patience = 50 epochs			

**Table 6:** Comparison of Surface Roughness Under Existing and DNN-Recommended Process Conditions for WAAM.

	Bead Geometry						Existing Working Conditions			Recommended Working Condition from the DNN		
Index	Thin Wall	Layer	θ*_L_* (°)	θ*_R_* (°)	Width (mm)	Height (mm)	Travel Speed (cm/min)	Feed Rate (cm/min)	Surface Roughness	Travel Speed (cm/min)	Feed Rate (cm/min)	Surface Roughness
1	1	2	102.11	101.94	6.95	2.06	31	560	3.255	30	540	3.133
2		3	100.98	99.87	6.91	3.73	31	560		30	570	
3		4	105.3	101.8	7.74	2.89	30	480		30	570	
4	9	2	97.07	97.24	6.01	2.87	30	480	3.459	30	560	2.939
5		3	101.6	101.73	7.72	2.74	33	650		30	540	
6		4	103.37	101.62	7.89	2.99	31	560		30	600	
7	10	2	97.08	96.81	6.81	2.87	31	560	3.522	30	540	3.219
8		3	105.03	104.75	7.54	2.6	31	560		30	570	
9		4	98.33	96.26	7.44	2.98	33	650		30	550	
10	14	2	101.33	100.13	7.52	2.9	30	480	4.015	30	570	3.058
11		3	100.72	98.31	6.55	2.95	31	560		30	540	
12		4	100.96	100.42	8.53	2.49	33	650		33	620	
13	22	2	102.99	103.25	7.49	2.97	30	480	3.396	30	500	2.986
14		3	99.64	98.28	6.73	2.66	31	560		30	540	
15		4	97.89	96.66	7.54	2.71	31	560		30	570	

**Table 7:** Statistical comparison of surface roughness between existing and recommended process conditions.

Thin Wall	Existing Surface Roughness	Recommended Surface Roughness	Improvement (µm)	Improvement (%)
1	3.255	3.133	0.122	3.7
9	3.459	2.939	0.52	15
10	3.522	3.219	0.303	8.6
14	4.015	3.058	0.957	23.8
22	3.396	2.986	0.410	12.1
Mean	3.529	3.067	0.462	13.1
*p*-value	0.0299 *			

* Significant at *p* < 0.05 (paired *t*-test).

## Data Availability

The original contributions presented in this study are included in the article. Further inquiries can be directed to the corresponding author.

## Abbreviations

The following abbreviations are used in this manuscript:

WAAM	Wire Arc Additive Manufacturing
AM	Additive Manufacturing
AI	Artificial Intelligence
GMAW	Gas Metal Arc Welding
VP-GTAW	Variable Polarity Gas Tungsten Arc Welding
GPR	Gaussian Process Regression
DMLS	Direct Metal Laser Sintering
GPRBOA	Gaussian Process Regression Surrogated Bayesian Optimization Algorithm
RL	Reinforcement Learning
CMM	Coordinate Measuring Machine
DNN	Deep Neural Network
MAPE	Mean Absolute Percentage Error
RMSE	Root Mean Squared Error
TP	True Positives
FP	False Positives
FN	False Negatives
ROC	Receiver Operating Characteristic
AUC	Area Under the Curve

## References

[1] Zhou Y., Chen H., Tang Y., Gopinath S., Xu X., Zhao Y.F. Simulation and Optimization Framework for Additive Manufacturing Processes. Proceedings of the 2014 International Conference on Innovative Design and Manufacturing (ICIDM).

[2] Dinovitzer M., Chen X., Laliberte J., Huang X., Frei H. (2019). Effect of Wire and Arc Additive Manufacturing (WAAM) Process Parameters on Bead Geometry and Microstructure. Addit. Manuf..

[3] Le V.T., Mai D.S., Tran V.C., Doan T.K., Balas V.E., Solanki V.K., Kumar R. (2021). Additive Manufacturing of Thin-Wall Steel Parts by Gas Metal Arc Welding Robot: The Surface Roughness, Microstructures and Mechanical Properties. Further Advances in Internet of Things in Biomedical and Cyber Physical Systems.

[4] Ziętala M., Durejko T., Polański M., Kunce I., Płociński T., Zieliński W., Łazińska M., Stępniowski W., Czujko T., Kurzydłowski K.J. (2016). The Microstructure, Mechanical Properties and Corrosion Resistance of 316L Stainless Steel Fabricated Using Laser Engineered Net Shaping. Mater. Sci. Eng. A.

[5] Lee S.H. (2020). Optimization of Cold Metal Transfer-Based Wire Arc Additive Manufacturing Processes Using Gaussian Process Regression. Metals.

[6] Busachi A., Erkoyuncu J., Colegrove P., Martina F., Ding J. (2015). Designing a WAAM Based Manufacturing System for Defence Applications. Procedia CIRP.

[7] So M.S., Seo G.J., Kim D.B., Shin J.-H. (2022). Prediction of Metal Additively Manufactured Surface Roughness Using Deep Neural Network. Sensors.

[8] Sommer K., Pfennig A., Sammler F., Abdelmoula M., Kamerer D., Heiler R. (2024). First Approach in Analysis of Tool Wear When Milling Additive Manufacturing (AM) Parts. Appl. Sci..

[9] Guo J., Li Q., Qin P., Yuan A., Lu M., Ke X., Zhang Y., Cheung B.C.F. (2025). Internal Surface Finishing and Roughness Measurement: A Critical Review. Chin. J. Aeronaut..

[10] Xiong J., Li Y., Li R., Yin Z. (2018). Influences of Process Parameters on Surface Roughness of Multi-Layer Single-Pass Thin-Walled Parts in GMAW-Based Additive Manufacturing. J. Mater. Process. Technol..

[11] Wang H., Jiang W., Ouyang J., Kovacevic R. (2004). Rapid Prototyping of 4043 Al-Alloy Parts by VP-GTAW. J. Mater. Process. Technol..

[12] Hanif M., Shah A.H., Shah I., Mumtaz J. (2023). Optimization of Bead Geometry during Tungsten Inert Gas Welding Using Grey Relational and Finite Element Analysis. Materials.

[13] Aiyiti W., Zhao W., Lu B., Tang Y. (2006). Investigation of the Overlapping Parameters of MPAW-based Rapid Prototyping. Rapid Prototyp. J..

[14] Li Y., Sun Y., Han Q., Zhang G., Horváth I. (2018). Enhanced Beads Overlapping Model for Wire and Arc Additive Manufacturing of Multi-Layer Multi-Bead Metallic Parts. J. Mater. Process. Technol..

[15] Ding D., Pan Z., Cuiuri D., Li H. (2015). A Multi-Bead Overlapping Model for Robotic Wire and Arc Additive Manufacturing (WAAM). Robot. Comput.-Integr. Manuf..

[16] Nguyen L., Buhl J., Bambach M. (2020). Multi-Bead Overlapping Models for Tool Path Generation in Wire-Arc Additive Manufacturing Processes. Procedia Manuf..

[17] Cao Y., Zhu S., Liang X., Wang W. (2011). Overlapping Model of Beads and Curve Fitting of Bead Section for Rapid Manufacturing by Robotic MAG Welding Process. Robot. Comput.-Integr. Manuf..

[18] Suryakumar S., Karunakaran K.P., Bernard A., Chandrasekhar U., Raghavender N., Sharma D. (2011). Weld Bead Modeling and Process Optimization in Hybrid Layered Manufacturing. Comput.-Aided Des..

[19] Calignano F., Manfredi D., Ambrosio E.P., Iuliano L., Fino P. (2013). Influence of Process Parameters on Surface Roughness of Aluminum Parts Produced by DMLS. Int. J. Adv. Manuf. Technol..

[20] Masood S.H., Rattanawong W., Iovenitti P. (2003). A Generic Algorithm for a Best Part Orientation System for Complex Parts in Rapid Prototyping. J. Mater. Process. Technol..

[21] Ning Y., Fuh J.Y.H., Wong Y.S., Loh H.T. (2004). An Intelligent Parameter Selection System for the Direct Metal Laser Sintering Process. Int. J. Prod. Res..

[22] Dong H., Cong M., Zhang Y., Liu Y., Chen H. Real Time Welding Parameter Prediction for Desired Character Performance. Proceedings of the 2017 IEEE International Conference on Robotics and Automation (ICRA).

[23] Wu Y., Kovacevic R. (2002). Mechanically Assisted Droplet Transfer Process in Gas Metal Arc Welding. Proc. Inst. Mech. Eng. Part B J. Eng. Manuf..

[24] Xiong J., Zhang G., Qiu Z., Li Y. (2013). Vision-Sensing and Bead Width Control of a Single-Bead Multi-Layer Part: Material and Energy Savings in GMAW-Based Rapid Manufacturing. J. Clean. Prod..

[25] Zhang H., Bai X., Dong H., Zhang H. (2024). Modelling and Prediction of Process Parameters with Low Energy Consumption in Wire Arc Additive Manufacturing Based on Machine Learning. Metals.

[26] Dharmawan A.G., Xiong Y., Foong S., Song Soh G. A Model-Based Reinforcement Learning and Correction Framework for Process Control of Robotic Wire Arc Additive Manufacturing. Proceedings of the 2020 IEEE International Conference on Robotics and Automation (ICRA).

[27] Wüst P., Edelmann A., Hellmann R. (2020). Areal Surface Roughness Optimization of Maraging Steel Parts Produced by Hybrid Additive Manufacturing. Materials.

[28] Song Y.-A., Park S., Choi D., Jee H. (2005). 3D Welding and Milling: Part I—A Direct Approach for Freeform Fabrication of Metallic Prototypes. Int. J. Mach. Tools Manuf..

[29] Mutua J., Nakata S., Onda T., Chen Z.-C. (2018). Optimization of Selective Laser Melting Parameters and Influence of Post Heat Treatment on Microstructure and Mechanical Properties of Maraging Steel. Mater. Des..

[30] Li W., Liu Z. (2011). A Method of SVM with Normalization in Intrusion Detection. Procedia Environ. Sci..

[31] Hinojosa Lee M.C., Braet J., Springael J. (2024). Performance Metrics for Multilabel Emotion Classification: Comparing Micro, Macro, and Weighted F1-Scores. Appl. Sci..

